# Tissue-adapted Tregs harness inflammatory signals to promote intestinal repair from therapy-related injury

**DOI:** 10.1038/s41392-025-02476-5

**Published:** 2025-11-26

**Authors:** Julius C. Fischer, Sascha Göttert, Maximilian Giller, Paul Heinrich, Kaiji Fan, Omer Khalid, Caroline N. Walther, Maria Drießlein, Sophie M. Nefzger, Gabriel Eisenkolb, Vincent R. Timnik, Sebastian Jarosch, Lena Klostermeier, Thomas Engleitner, Nicholas Strieder, Claudia Gebhard, Sarah Diederich, Nicole A. Schmid, Laura Lansink Rotgerink, Laura Joachim, Sakhila Ghimire, Eva Vonbrunn, Maike Büttner-Herold, Marianne Remke, Katja Steiger, Rupert Öllinger, Roland Rad, Daniel Wolff, Markus Feuerer, Petra Hoffmann, Matthias Edinger, Michael Rehli, Markus Tschurtschenthaler, Oliver Kepp, Guido Kroemer, Erik Thiele Orberg, Stephanie E. Combs, Wolfgang Herr, Florian Bassermann, Dirk H. Busch, Ernst Holler, Simon Heidegger, Hendrik Poeck

**Affiliations:** 1 Department of Radiation Oncology, Technical University of Munich (TUM), TUM School of Medicine and Health, TUM University Hospital, Munich, Germany; 2 Centre de Recherche des Cordeliers, Equipe labellisée par la Ligue contre le cancer, Université Paris Cité, Sorbonne Université, Inserm U1138, Institut Universitaire de France, Paris, France; 3 Metabolomics and Cell Biology Platforms, Gustave Roussy Cancer Center, Université Paris Saclay, Villejuif, France; 4 Department for Internal Medicine III, Technical University of Munich (TUM), TUM School of Medicine and Health, TUM University Hospital, Munich, Germany; 5 Technical University of Munich, TUM School of Medicine and Health, Center for Translational Cancer Research (TranslaTUM), Munich, Germany; 6 University Hospital Regensburg, Department for Internal Medicine III, Hematology & Oncology, Regensburg, Germany; 7 Leibniz Institute for Immunotherapy (LIT), Regensburg, Germany; 8 Department of Nephrology, Technical University Munich (TUM), TUM School of Medicine and Health, TUM University Hospital, Munich, Germany; 9 Department of Gynecology and Obstetrics, Technical University of Munich (TUM), TUM School of Medicine and Health, TUM University Hospital, Munich, Germany; 10 Technical University of Munich (TUM), TUM School of Medicine and Health, Institute for Medical Microbiology, Immunology and Hygiene, Munich, Germany; 11 Technical University of Munich (TUM), TUM School of Medicine and Health, Institute of Molecular Oncology and Functional Genomics, Munich, Germany; 12 Department of Nephropathology, Institute of Pathology, Universitätsklinikum Erlangen, Friedrich-Alexander-Universität Erlangen-Nürnberg (FAU), Erlangen, Germany; 13 Technical University of Munich (TUM), TUM School of Medicine and Health, Comparative Experimental Pathology, Munich, Germany; 14 Technical University of Munich (TUM), TUM School of Medicine and Health, Institute of Pathology, Munich, Germany; 15 German Cancer Consortium (DKTK), partner site Munich, A partnership between DKFZ and TUM University Hospital, Munich, Germany; 16 Department of Medicine II, Technical University of Munich (TUM), TUM School of Medicine and Health, TUM University Hospital, Munich, Germany; 17 Technical University of Munich, TUM School of Medicine and Health, TUM University Hospital, Chair of Translational Cancer Research and Institute of Experimental Cancer Therapy, Munich, Germany; 18 Institut du Cancer Paris CARPEM, Hôpital Européen Georges Pompidou, France-HP, Paris, France; 19 Bavarian Cancer Research Centre (BZKF), Munich, Germany; 20 Bavarian Cancer Research Centre (BZKF), Regensburg, Germany

**Keywords:** Bone marrow transplantation, Inflammation, Gastrointestinal diseases, Intestinal stem cells

## Abstract

Intestinal stem cells (ISCs) promote tissue repair after genotoxic or immune-mediated injury. However, ISCs are particularly sensitive to various stressors and primary targets of overwhelming immune responses, such as interferon γ (IFNγ)-mediated killing. In mouse models of radiation therapy-induced gut damage and in biopsies from patients who underwent allogeneic hematopoietic stem cell transplantation, we observed IFNγ expression by intestinal T_reg_ cells. T_reg_ cells leverage combined IFNγ and interleukin 10 (IL-10) stimulation of ISCs to nurture the growth of intestinal organoids through the activation of the mTORC1 and Myc pathways. Similarly, T_reg_ cells or the combined addition of recombinant IFNγ and IL-10 promoted the regeneration of organoids after irradiation, and both cytokines were essential for ensuring epithelial regeneration following acute intestinal tissue injury in vivo. The exposure of organoids to growth factor-free culture conditions revealed distinct EGF-like properties of IFNγ and Wnt-like properties of IL-10. While IFNγ rapidly induced epithelial proliferation, it depleted the pool of ISCs in vitro. Only the combination of IFNγ and IL-10 led to epithelial proliferation and organoid growth while simultaneously ensuring ISC maintenance over time. Our results reveal a context-dependent role of inflammatory signaling in ISCs, through which T_reg_ cells promote epithelial repair following therapy-induced injury.

## Introduction

Therapy-induced tissue injury represents a major challenge in oncology, limiting the efficacy and tolerability of many cancer treatments. The intestine is particularly vulnerable to damage from systemic therapies such as chemotherapy and immunotherapy, as well as from local interventions targeting tumors adjacent to or within the gastrointestinal tract (for example, radiation therapy).^1,2^ Both direct cytotoxic effects on epithelial and stromal cells and indirect immunological mechanisms, including cytokine-mediated inflammation, disruption of barrier homeostasis, and dysregulated immune activation, contribute to the pathogenesis of intestinal injury and related side effects.^1–3^ As gastrointestinal toxicity remains a major dose-limiting factor for numerous anticancer therapies, there is an urgent need for novel approaches that integrate experimental models with clinical observation to unravel these interconnected mechanisms and guide the development of targeted strategies to prevent and mitigate severe intestinal adverse events.

Regulatory T (T_reg_) cells are a specialized subset of CD4⁺ T cells that play a central role in maintaining immune tolerance and preventing excessive immune activation.^4^ Defined by the transcription factor Forkhead box P3 (Foxp3)^+^, T_reg_ cells exert potent immunosuppressive functions through several mechanisms, including the secretion of inhibitory cytokines such as IL-10 and TGF-β, metabolic disruption of effector T cells, and modulation of antigen-presenting cell activity.^4,5^ By controlling the magnitude and duration of immune responses, T_reg_ cells prevent autoimmune reactions and limit immunopathology during infection and inflammation. Their ability to suppress a broad range of immune cell types, including conventional T cells, B cells, and innate immune populations, positions them as key regulators of immune equilibrium.^4–6^ Dysregulation or functional impairment of T_reg_ cells is associated with autoimmunity, chronic inflammation, and other immune-mediated diseases, emphasizing their indispensable role in maintaining immunological balance.^4^ In addition to these immunosuppressive functions, T_reg_ cells also support tissue homeostasis and repair.^7–12^ However, their regenerative functions are tissue- and context-dependent, and the underlying mechanisms remain poorly understood.^4,8,13,14^ Nonetheless, adoptive T_reg_ cell transfer has been proposed as a strategy to alleviate immunopathogenic diseases, including graft-versus-host disease (GVHD), which is a serious complication of allogeneic hematopoietic stem cell transplantation (allo-HSCT).^15,16^ GVHD pathogenesis is initiated by the effects of pretransplant conditioning therapy, which causes tissue damage and inflammation.^16^ This leads to alloreactive priming, expansion, and excessive effector function of donor T cells, ultimately resulting in GVHD.^17^ Several studies have demonstrated that maintaining intestinal epithelial barrier function after conditioning therapy and promoting intestinal stem cell (ISC)-driven epithelial repair can limit the development of severe GVHD.^18–21^ More broadly, intestinal epithelial damage is a common side effect of cancer therapies, including chemotherapy, immunotherapy, and radiation therapy. Thus, promoting epithelial regeneration represents an important therapeutic strategy.^1,3,22^

Intestinal epithelial function and the stem cell niche, including ISC properties, can be studied in three-dimensional multicellular organoid cultures.^23–25^ Organoids have recently revealed how different T-cell subsets and their cytokines affect ISC renewal and differentiation, thereby contributing to epithelial regeneration after injury.^18,20,25–28^ Among other functions, T_reg_ cells and their key cytokine IL-10 have been shown to help maintain the ISC niche and its stem-like function.^27^ This contrasts with other T helper cell effector cytokines, which reduce ISC renewal and promote ISC differentiation.^27^ In the context of tissue injury, recent studies in mice revealed that allogeneic T cells invade intestinal tissue and epithelial crypts early after allogeneic bone marrow transplantation (allo-BMT) and kill ISCs via epithelial IFNγ receptor (IFNγR) activation.^29,30^ This may explain previous studies linking IFNγ to aggravated GVHD and epithelial tissue damage in infectious and autoinflammatory colitis.^26,31–42^ However, GVHD is worsened in allo-BMT recipients receiving IFNγ-deficient donor T cells.^43–49^ Thus, the role of IFNγ in intestinal injury remains controversial, and reconciling these seemingly conflicting findings has been a longstanding challenge.^26,29,31–41,43–55^

Here, we report that the infiltration of IFNγ-producing T cells into the intestinal epithelium contributes to both intestinal damage and subsequent regeneration. Single-cell RNA sequencing (scRNA-seq) analyses of murine and human intestinal T_reg_ cells revealed IFNγ expression specifically after tissue adaptation upon intestinal injury. T_reg_ cells promote repair by orchestrating concomitant IFNγ and IL-10 signaling in intestinal epithelial cells, thereby skewing toxic IFNγ effects toward regenerative signals. scRNA-seq identified that T_reg_ cells and simultaneous IFNγ/IL-10 stimulation activate mTORC1 and *Myc* signaling in ISCs, thereby promoting regeneration. Our data identify a hitherto undefined mechanism whereby tissue-adapted T_reg_ cells integrate inflammatory signals to directly foster intestinal tissue repair. These results open opportunities to selectively promote regenerative immune signaling to limit intestinal side effects. Targeting this mechanism could prevent excessive tissue damage while supporting mucosal recovery.

## Results

### Excessive intestinal tissue injury and regeneration are linked by IFNγ and Treg cells

**Fig. 1:**
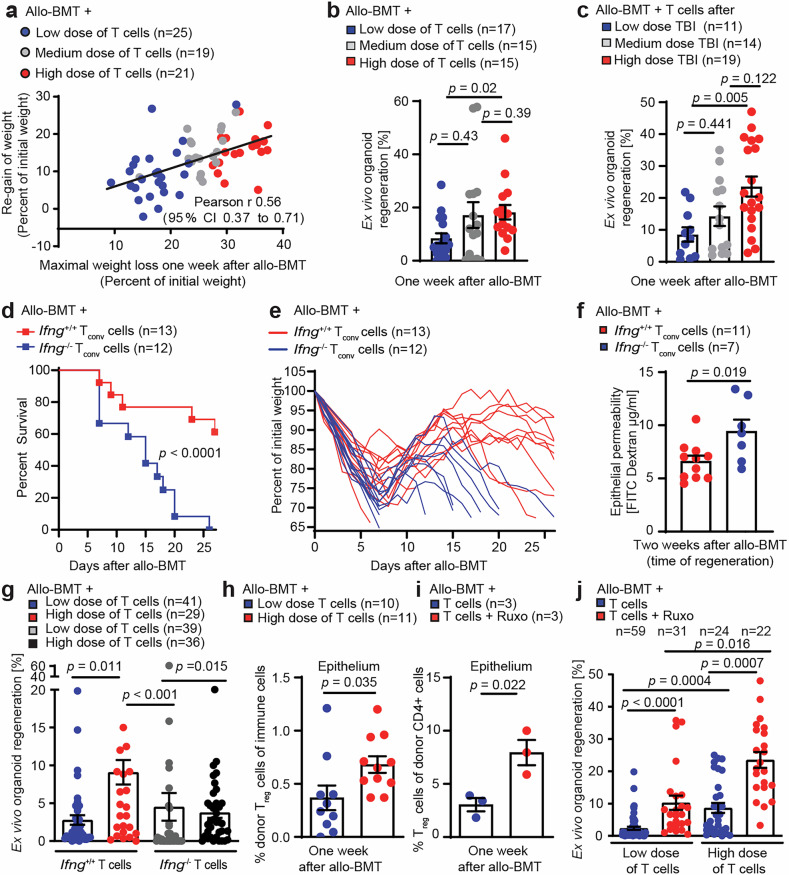
Excessive intestinal tissue injury and regeneration are linked by IFNγ and T_reg_ cells. **a** BALB/c mice received 9 Gy TBI followed by allo-BMT (C57BL/6 J donors) of BM ± allogeneic T cells (low dose: 0.1 × 10^6 ^T cells; medium dose: 0.5 × 10^6 ^T cells; high dose: 2.5 × 10^6 ^T cells). Correlation between maximum weight loss (day 7) and peak weight recovery 2 weeks after allo-BMT. The data were pooled from 3 independent experiments. **b** Ex vivo small intestine (SI) organoid regeneration rate ( = number of established organoids on day 4 after starting ex vivo organoid culture divided by the number of cultured crypts, see Fig. S1f for details) on day 7 after allo-BMT in mice co-transplanted with increasing numbers (low, medium, high) of allogeneic T cells or (**c**) increasing doses of TBI conditioning prior to allo-BMT with a medium dose of T cells. The data were pooled from 3 independent experiments. **d** Survival and **e** weight loss of individual mice after they received TBI (9 Gy) followed by allo-BMT (C57BL/6 J donors) with *Ifng*^+/+^ BM and *Ifng*^+/+^ or *Ifng*^−/−^ T_conv_ cells (C57BL/6 J donors). Pooled data from 2 independent experiments. **f** Intestinal FITC-dextran permeability assay two weeks (d16) after allo-BMT. Pooled data from 3 experiments. **g** Allo-BMT with WT BM and a low or high dose of *Ifng*^+/+^ or *Ifng*^−/−^ T cells. Ex vivo SI organoid regeneration rate. Pooled data from 4 experiments. **h** SI intraepithelial leukocytes were isolated one week after allo-BMT and analyzed via flow cytometry after 4 h of restimulation in vitro. Frequency of FoxP3^+^ CD4^+^ donor (H-2K^b+^) T_reg_ cells among all live CD45^+^ immune cells. **i** Allo-BMT with BM and 1 × 10^6 ^T cells ± ruxolitinib treatment (30 mg/kg body weight, administered orally twice daily from day –1 prior to allo-BMT until the day before analysis). The intraepithelial donor T_reg_ cells are depicted on day 7 after allo-BMT. **j** Allo-BMT with BM and a low or high dose of T cells ± ruxolitinib treatment from d-1 onward (30 mg/kg body weight, administered orally twice daily). Ex vivo SI organoid regeneration rate on day 7 after allo-BMT. Pooled data from 4 independent experiments. The data of the control group, which included a low dose of T cells and a high dose of T cells, are also shown in Fig. 1g. The data in Fig. 1b, c, f–j are presented as the means ± S.E.M.s and were analyzed via unpaired two-tailed t tests or Kruskal‒Wallis tests (Fig. 1b, c, g, j) with Dunn’s multiple comparisons test for multiple comparisons. The number of biological replicates (*n*), indicating the number of mice analyzed, is shown in the figure for all individual experiments

To investigate the relationship between damage severity and subsequent regeneration of the ISC compartment, we performed murine allo-BMTs with varying numbers of co-transplanted allogeneic T cells. The mice that experienced the most prominent acute weight loss following BMT also presented the greatest potential for weight recovery after passing the nadir (Fig. S1a, b), as indicated by the positive correlation between the severity of initial weight loss and the extent of subsequent clinical recovery (Fig. 1a). Immunohistochemical analyses and bulk transcriptomic profiling of small intestinal tissue one week after allo-BMT revealed that co-transplantation of T cells dose-dependently increased intestinal T-cell homing, activation, and disease activity (Fig. S1c, d, Tables S1–3) but also induced genes related to tissue repair (e.g., type I interferon signatures and tryptophan metabolism) (Fig. S1e, Tables S1, 2).^20,56^ Thus, the highest dose of allogeneic T cells caused the most pronounced weight loss, followed by the strongest regenerative response.

Given the fundamental role of the ISC niche in promoting intestinal healing, we next analyzed the capacity of the ISC compartment to support regeneration after injury (assessed by the percentage of isolated crypts that survive and form organoids during in vitro culture) (Fig. S1f). We observed that co-transplantation of greater numbers of allogeneic T cells resulted in enhanced ex vivo regeneration of small intestine (SI) organoids (Fig. 1b). Similarly, increasing intestinal T-cell infiltration by increasing the conditioning dose of total body irradiation (TBI) (Fig. S1g) enhanced ex vivo regeneration of SI organoids (Fig. 1c).

Intestinal infiltration and effector function (e.g., IFNγ expression) of conventional T (T_conv_) cells are hallmarks of GVHD pathogenesis and are linked to increased mortality.^16^ We therefore performed allo-BMTs using *Ifng*^−/−^ donors. Compared with those of mice co-transplanted with *Ifng*^*+/+*^ T_conv_ cells, the survival of allo-BMT recipients co-transplanted with *Ifng*^*−/−*^ T_conv_ cells was strongly reduced (Fig. 1d). Indeed, the recipients of *Ifng*^−/−^ T_conv_ cells failed to recover from acute injury during the second week after allo-BMT (Fig. 1e). Consistently, we observed a significant reduction in intestinal epithelial integrity (as indicated by enhanced FITC-dextran translocation from the gut lumen to the bloodstream) two weeks after allo-BMT with the co-transfer of *Ifng*^−/−^ T cells (Fig. 1f). Next, we studied ex vivo SI organoid regeneration in allo-BMT recipients co-transplanted with varying numbers of *Ifng*^+/+^ or *Ifng*^−/−^ T cells to explore the contribution of IFNγ to intestinal regeneration. Unlike mice co-transplanted with high doses of *Ifng*^+/+^ T cells, those receiving high doses of *Ifng*^−/−^ T cells failed to exhibit an increase in ex vivo organoid regeneration (Fig. 1g).

In summary, our findings suggest that IFNγ expression by infiltrating intestinal T cells supports ISC niche regeneration after injury.

We next characterized intestinal T-cell infiltration after allo-BMT and discovered that mice transplanted with high numbers of T cells presented an increased abundance of allogeneic T_reg_ cells at the peak of intestinal tissue injury (Figs. 1h, S1h). The Janus kinase (JAK1 and JAK2) inhibitor ruxolitinib reportedly promotes T_reg_ differentiation and ameliorates GVHD.^57–59^ Accordingly, treatment with ruxolitinib increased the frequency of epithelial T_reg_ cells following allo-BMT (Figs. 1i, S1h, i), reduced acute GVHD morbidity (Fig. S1j), and enhanced ex vivo organoid regeneration. This regenerative effect of ruxolitinib was independent of the number of co-transplanted T cells but was greatest in the mice that received high numbers of allogeneic T cells (Fig. 1j).

Collectively, these results demonstrate that (i) gut-infiltrating IFNγ-expressing T cells contribute to epithelial regeneration after allo-BMT and (ii) that increased epithelial T_reg_ cell frequencies are linked with improved regeneration of the ISC niche. By combining both aspects, we hypothesized that gut-invading and IFNγ-producing T_reg_ cells might stimulate intestinal regeneration.

### T_reg_ cells adapt to intestinal epithelial tissue injury via IFNγ expression

**Fig. 2:**
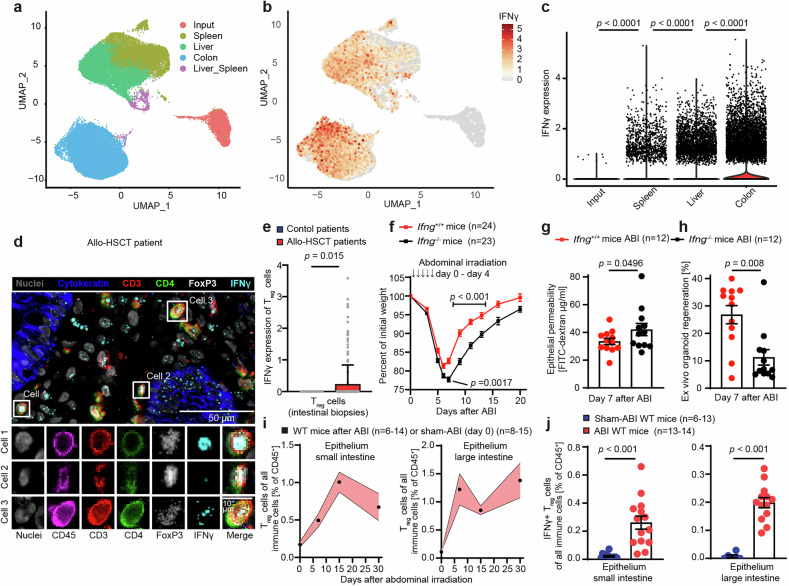
T_reg_ cells adapt to intestinal epithelial tissue injury via IFNγ expression. **a** A published scRNA-seq dataset (GEO accession number: GSE223798) was used.^60^ Briefly, donor T_reg_ cells were expanded in vitro, analyzed (input T_reg_ cells) or supplied to recipient mice and then extracted from the respective tissues. Plots of single cells in UMAP space for all experimental conditions, colored by their origin or **b** colored by their IFNγ expression (library size normalized and log-transformed UMI counts). **c** IFNγ expression (library-size normalized and log-transformed UMI counts) in tissue and input T_reg_ cells (Input *n* = 3068, Colon *n* = 14,862, Liver *n* = 10,153, Spleen *n* = 8606, 3 mice). Differential expression testing was performed with the Wilcoxon test, and the resulting *p*-values were corrected for multiple testing with the Bonferroni method. **d** Exemplary image of chip cytometry of intestinal biopsies of human allo-HSCT recipients. Upper image: overlay of all indicated markers in a larger area (scale bar = 50 µm). Lower images: high-resolution images of single markers and their overlay for three representative single cells (scale bar = 10 µm). **e** IFNγ expression (library-size normalized UMI counts) of T_reg_ cells was analyzed via scRNA-seq of cells isolated from large intestinal biopsies of allo-HCST recipients (*n* = 22 patients, with *n* = 208 identified T_reg_ cells) or control patients who did not undergo allo-HSCT (*n* = 5 patients with *n* = 34 identified T_reg_ cells). **f** C57BL/6 J *Ifng*^+/+^ or *Ifng*^−/−^ mice received abdominal irradiation (ABI, 5 × 4.5 Gy/day from day 0 until day 4), and body weight was monitored. Pooled data from 2 experiments. Statistical comparisons are shown for the day of weight nadir (day 6) and for days 9, 11, and 13 of the recovery phase. **g** Intestinal FITC-dextran permeability assay on day 7 following ABI (5 × 4.5 Gy/day). Pooled data from 2 independent experiments. **h** Ex vivo SI organoid regeneration rate on day 7 following ABI in *Ifng*^+/+^ or *Ifng*^−/−^ mice. Pooled data from 2 experiments. **i** SI and large intestine (LI) intraepithelial leukocytes were analyzed by flow cytometry. The cells were isolated at different time points (7, 15, and 30 days) following ABI of the C57BL/6 J WT mice. Control mice without an ABI were pooled and are shown as day 0 after ABI. The graphs show the percentages of T_reg_ cells among all live CD45^+^ immune cells. Pooled data from 7 experiments. **j** Graph showing the percentage of IFNγ^+^ T_reg_ cells among all live CD45^+^ intraepithelial leukocytes in C57BL/6 J WT mice on day 15 after the start of ABI, as analyzed by flow cytometry after in vitro restimulation. The data were pooled from 3 independent experiments. The data are presented as the means ± S.E.M.s and were analyzed via unpaired two-tailed t tests, Mann‒Whitney tests (**e**, **h**), or ordinary one-way ANOVA (with Dunn’s multiple comparisons test) for multiple comparisons. The data presented in (**g**) were analyzed via an unpaired one-tailed *t* test on the basis of the hypothesis of increased injury in *Ifng*^−/−^ mice, as suggested by the results shown in (**f**). The number of biological replicates (*n*), indicating the number of mice analyzed, is shown in the figure for all individual experiments

The adoptive transfer of donor Treg cells is a promising strategy for the prevention of GVHD. A recent study discovered that T_reg_ cells that were injected into mice and then recovered from different tissues (spleen, liver, and large intestine) acquired organ-specific gene expression profiles within one week after allo-BMT.^60^ We re-analyzed the corresponding scRNA-seq data (Fig. 2a) and discovered that T_reg_ cells isolated from the intestine but not input T_reg_ cells (and less so T_reg_ cells isolated from the spleen or liver) noticeably expressed IFNγ mRNA (Fig. 2b, c). We performed allo-BMTs with co-transplanted T cells and then employed immunofluorescence cytometry of single-cell suspensions to confirm IFNγ expression by donor T_reg_ cells in the intestinal epithelium and lamina propria one week after allo-BMT (Fig. S2a, b).

We next analyzed T_reg_ cells in gastrointestinal tissue biopsies from human allo-HSCT recipients via chip cytometry^61,62^ and identified T_reg_ cells with varying levels of IFNγ expression in close proximity to the intestinal epithelium (Fig. 2d). Comparative analyses of scRNA-seq data from allo-HSCT recipients and control patients confirmed the presence of intestinal T_reg_ cells with similar FOXP3 expression levels (Fig. S2c). However, IFNγ expression was detectable only in intestinal T_reg_ cells from allo-HSCT recipients but not in T_reg_ cells from control patients (Fig. 2e). Thus, in human patients, allo-HSCT is also associated with the reprogramming of intestinal T_reg_ cells toward IFNγ production.

The pathogenesis of GVHD following allo-BMT is complex, as it is initiated by cytotoxic pre-transplant conditioning and exacerbated by allogeneic T-cell responses. To generalize our findings, we employed a simpler model of intestinal damage relying on abdominal irradiation (ABI), which directly damages the intestinal epithelium. Compared with *Ifng*^*+/+*^ control mice, ABI-treated *Ifng*^−/−^ mice presented more severe radiogenic enteritis, as demonstrated by increased acute weight loss, impaired intestinal epithelial integrity, and prolonged weight loss (Fig. 2f, g). Moreover, we detected reduced ex vivo SI organoid regeneration in *Ifng*^−/−^ mice following ABI (Fig. 2h). As this occurs after allo-BMT, ABI leads to increased T_reg_ cell abundance in both large and small intestinal tissues, accompanied by elevated IFNγ expression (Figs. 2I, j, S2d–g).

We conclude that IFNγ expression by T_reg_ cells occurs after intestinal tissue damage and may subsequently contribute to epithelial regeneration.

### T_reg_ cell-mediated intestinal organoid growth requires epithelial IFNγ-receptor signaling

**Fig. 3:**
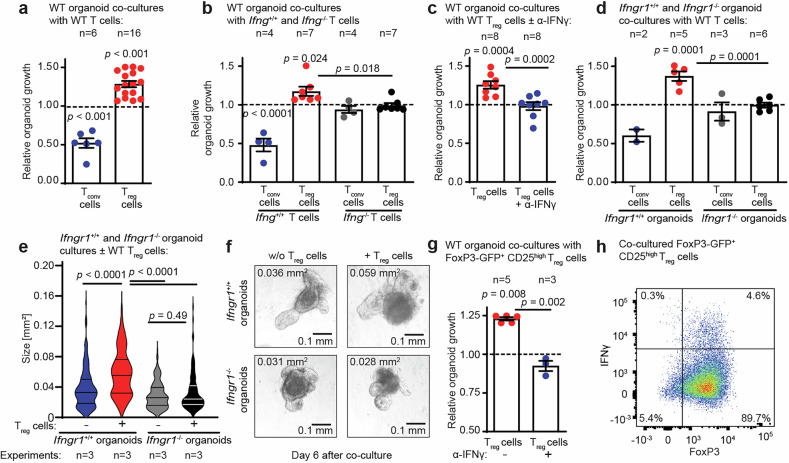
T_reg_ cell-mediated intestinal organoid growth requires epithelial IFNγ receptor signaling. **a** Relative organoid growth of murine SI organoids co-cultured with allogeneic and stimulated (IL-2 + beads) CD4^+^CD25^-^ T_conv_ cells or CD4^+^CD25^+^ T_reg_ cells MACS-isolated from splenocytes of WT C57BL/6 J mice (detailed description of “relative organoid growth” in the methods section and Fig. S3a). The dotted line represents the growth of control organoids cultured without any T cells. **b** Relative organoid growth of murine SI organoids co-cultured with allogeneic T_conv_ or T_reg_ cells isolated from *Ifng*^+/+^ or *Ifng*^−/−^ mice. Relative organoid growth of murine SI organoids co-cultured with **c** allogeneic WT T_reg_ cells in the presence of IFNγ blocking antibody (anti-IFNγ = α-IFNγ) (control organoids were cultured without any T cells but were also stimulated with IL-2 and α-IFNγ) **d** Murine SI organoids isolated from *Ifngr1*^+/+^ or *Ifngr1*^−/−^ mice (C57BL/6 J) were co-cultured with allogeneic WT T_reg_ cells (BALB/c) to analyze relative organoid growth and **e** mean size (area) after co-culture. Area of murine SI organoids on day 6 after co-culture; number of measured organoids: untreated *Ifngr1*^+/+^ (*n* = 167) and *Ifngr1*^−/−^ (*n* = 163) organoids; *Ifngr1*^+/+^ (*n* = 116) and *Ifngr1*^−/−^ (*n* = 113) organoids with T_reg_ cells. **f** Exemplary images (converted to grayscale) of organoids after co-culture. **g** Relative murine SI organoid growth after co-culture with flow cytometry-sorted (CD4^+^ CD25^hi^ eGFP-Foxp3^+^) T_reg_ cells ± α-IFNγ (left panel), and **h** IFNγ expression of T_reg_ cells after removal from co-culture on day 4 and 4 h restimulation (eBioscience™ Cell Stimulation Cocktail plus protein transport inhibitors) (right panel). Violin plots (**e**) showing the distribution of values with medians and quartiles indicated. All other data are presented as the mean ± S.E.M. and were analyzed via an unpaired two-tailed t test or ordinary one-way ANOVA (with Dunn’s multiple comparisons test) for multiple comparisons. The number of biological replicates (*n*), indicating the number of separate organoid culture experiments, is shown in the figure for all individual experiments

To explore the role of IFNγ in the interaction between intestinal epithelial cells and different T-cell subsets, including T_reg_ cells, we co-cultured intestinal organoids with T cells and then assessed the number of developing organoids after the first passage (Fig. S3a). Allogeneic wild-type (WT) CD4^+^CD25^-^ conventional T (T_conv_) cells impaired SI organoid growth. In contrast, WT CD4^+^CD25^+^ T_reg_ cells enhanced organoid growth (Fig. 3a). Consistent with prior studies demonstrating the toxic effects of IFNγ on ISCs, IFNγ-deficient (*Ifng*^−/−^) T_conv_ cells failed to reduce organoid counts (Fig. 3b).^26,29^ Intriguingly, T_reg_ cell-promoted organoid growth was also IFNγ dependent, as *Ifng*^–/–^ T_reg_ cells failed to increase organoid counts (Fig. 3b). Moreover, the beneficial effect of T_reg_ cells on organoid growth was abolished by neutralizing anti-IFNγ antibodies (Fig. 3c).

*Ifngr1*^–/–^ T_reg_ cells (which cannot respond to IFNγ owing to the absence of the IFNγ receptor) retained their ability to promote organoid growth (Fig. S3b). In contrast, *Ifngr1*^–/^– epithelial cells failed to increase their growth in response to WT T_reg_ cells (Fig. 3d), indicating that the IFNγ receptor must operate in epithelial cells, not T_reg_ cells, to stimulate SI organoid growth. Moreover, co-culture with *Ifng*^+/+^ T_reg_ cells increased the size of developing organoids, which was dependent on epithelial IFNγR activation (Fig. 3e, f). This effect on organoid size was also lost when WT T_reg_ cells were replaced with *Ifng*^–/–^ T_reg_ cells or when IFNγ was neutralized by antibodies (Fig. S3c). When we increased the stringency of T_reg_ purification (by flow cytometric purification of CD4^+^CD25hi FoxP3-eGFP^+^ cells), we continued to observe IFNγ-dependent SI organoid growth stimulation by T_reg_ cells. This occurred in spite of the fact that only ~5% of Tregs contained immunofluorescence-detectable IFNγ (Fig. 3g, h).

In summary, IFNγ plays a dual role in the intestinal response to stress. IFNγ produced by T_conv_ cells drives epithelial damage, whereas IFNγ produced by T_reg_ cells apparently promotes intestinal organoid growth.

### T_reg_ cell-mediated IL-10 and IFNγ co-stimulation promotes murine and human intestinal organoid growth and repair from injury

**Fig. 4:**
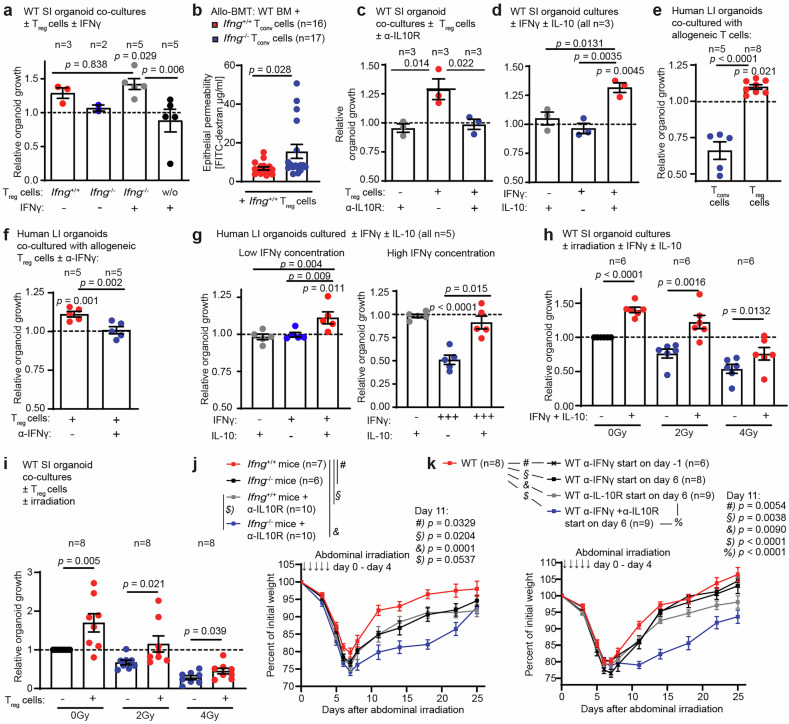
T_reg_ cell-mediated IL-10 and IFNγ co-stimulation promotes murine and human intestinal organoid growth and repair from injury. **a** Relative growth of murine SI WT organoids co-cultured with allogeneic T_reg_ cells isolated from *Ifng*^+/+^ or *Ifng*^−/−^ mice ± stimulation with 0.25 ng/mL rIFNγ. The dotted line represents the growth of control organoids cultured without any T cells. **b** Allo-BMT with WT BM plus WT T_reg_ cells and *Ifng*^+/+^ or *Ifng*^−/−^ T_conv_ cells. Intestinal FITC-dextran permeability assay 2 weeks (d16) after allo-BMT. Pooled data from 3 experiments**. c** Relative organoid growth of murine SI WT organoids co-cultured with allogeneic WT T_reg_ cells ± anti-IL-10R antibody (α-IL10R) or **d** stimulated with ± 10 ng/mL rIL-10 and ± 0.25 ng/mL rIFNγ in the absence of T_reg_ cells. Relative organoid growth of human patient-derived LI organoids co-cultured with **e** allogeneic T_conv_ or T_reg_ cells, **f** with allogeneic T_reg_ cells ± α-IFNγ, or **g** stimulated ± rIL-10 ± rIFNγ. Left panel: rIFNγ 0.25 ng/mL; right panel: rIFNγ 5 ng/mL. **h** Relative growth of murine SI organoids after in vitro irradiation ± cytokine stimulation or **i**) subsequent co-culture with syngeneic T_reg_ cells. **j** *Ifng*^+/+^ or *Ifng*^−/−^ mice received ABI (5 × 4.5 Gy/day from day 0 until day 4), and body weight was monitored (treatment schedule is depicted in Fig. S4h; α-IL10-R injections on day-1, day 3, 7, 10, and 14). Pooled data from 2 experiments. **k** WT mice received ABI (5×4.5 Gy/day from day 0 until day 4) ± α-IFNγ ± α-IL-10R at the indicated time points (the treatment schedule is depicted in Fig. S4j; group day 1: injections on days 1, 3, 6, 9, 13, and 16; group day 6: injections on days 6, 9, 13, and 16). Pooled data from 2 experiments. Body weight during regeneration (day 11) was analyzed via ordinary one-way ANOVA plus Fisher’s LSD. The data were analyzed via two-tailed or one-tailed methods (**i**, on the basis of the hypothesis of improved T_reg_-mediated regeneration, as suggested by the results shown in Fig. 3a, (**d**, **h**). Unpaired t-test or ordinary one-way ANOVA for multiple comparisons (with Dunn’s multiple comparisons test) and are presented as the means ± S.E.M. The number of biological replicates (*n*), indicating the number of separate organoid culture experiments or the number of mice analyzed, is shown in the figure for all individual experiments

Next, we measured IFNγ concentrations in vitro and found that T_reg_ cells produced low levels of IFNγ when cocultured with SI organoids (Fig. S4a). Notably, such low doses of recombinant (r)IFNγ did not affect organoid growth, although they restored the ability of *Ifng*^−/−^ T_reg_ cells to induce intestinal organoid growth in vitro, suggesting that IFNγ is not the sole factor produced by T_reg_ cells that contributes to intestinal repair (Fig. 4a). We observed significantly increased epithelial permeability during the recovery phase in mice co-transplanted with *Ifng*^−/−^ T_conv_ cells and *Ifng*^+/+^ T_reg_ cells compared with that in recipients of both *Ifng*^*+/+*^ T_conv_ and T_reg_ cells (Fig. 4b). Reduced epithelial barrier function in allo-BMT recipients co-transplanted with *Ifng*^−/−^ T_conv_ cells and *Ifng*^+/+^ T_reg_ cells was associated with significantly reduced survival (Fig. S4b). We concluded that non-T_reg_ cell-derived IFNγ (T_conv_ cell IFNγ) is indeed important for tissue regeneration during allo-BMT. Together with our in vitro finding that rIFNγ was not sufficient to promote organoid growth in the absence of T_reg_ cells (Fig. 4a), these data suggest that T_reg_ cells need to provide at least one additional signal along with epithelial IFNγR signaling for successful intestinal recovery after allo-BMT.

Seeking this complementary T_reg_ cell signal, we found that following allo-BMT, T_reg_ cells adapt toward IL-10 expression upon tissue adaptation but independent of the invaded tissue side (Fig. S4c). This finding contrasts with the IFNγ expression observed predominantly in T_reg_ cells after intestinal tissue adaptation (Figs. 2a–c, S4d). Consistently, we also observed an increase in the fraction of IL-10–expressing T_reg_ cells following intestinal irradiation, revealing that this model also shows an increase in both IFNγ- and IL-10–expressing T_reg_ cells after intestinal injury (Fig. S4e, f). Hypothesizing that IL-10 serves as the secondary signal supporting regeneration in IFNγR-activated SI organoids, we found that blocking IL-10 receptor signaling completely abolished the organoid growth-promoting effect of T_reg_ cells (Fig. 4c). Stimulation of intestinal organoids with the combination of rIFNγ and rIL-10 but not with either single cytokine alone was sufficient to induce organoid growth (Fig. 4d).

Next, we aimed to confirm our findings in a human organoid model. Therefore, we established an in vitro allogeneic co-culture of patient-derived intestinal organoids (large intestine) and T-cell subsets isolated from unrelated healthy volunteers (Fig. S4g). We found that human T_conv_ cells decreased the number of co-cultured large intestine (LI) organoids, whereas T_reg_ cells promoted organoid growth (Fig. 4e). In line with our murine data, T_reg_ cell-mediated organoid growth was completely dependent on IFNγ (Fig. 4f). Moreover, only the combined addition of rIL-10 and rIFNγ (but not that of either cytokine alone) induced human LI organoid growth. In addition, high rIFNγ doses resulted in direct organoid toxicity that was largely counterbalanced by co-supplementation with rIL-10 (Fig. 4g).

Next, we characterized the role of IL-10 and IFNγ in non-T-cell-mediated cytotoxicity and tissue regeneration by analyzing intestinal organoids after irradiation in vitro. As expected, irradiation resulted in a dose-dependent reduction in organoid growth (Fig. 4h). Simultaneous stimulation with IFNγ and IL-10 enhanced organoid regeneration despite exposure to irradiation (Fig. 4h). A similar effect was observed when irradiated organoids were co-cultured with syngeneic T_reg_ cells (Fig. 4i).

In conclusion, T_reg_ cells can promote murine and human intestinal organoid growth via the combined effects of IFNγ and IL-10. Through this mechanism, T_reg_ cells stimulate recovery from immune-mediated and radiation-induced intestinal injury. However, since combined stimulation with recombinant cytokines promoted organoid growth, our data suggest that this effect can, in principle, occur independently of the cytokines’ cellular origin. To further delineate the synergistic effect of both cytokines during intestinal regeneration in vivo, we performed ABI in *Ifng*^*−/−*^ and *Ifng*^*+/+*^ controls, with or without additional IL-10R blockade starting prior to irradiation (Fig. S4h). We observed increased acute weight loss and delayed recovery in both *Ifng*^*−/−*^ and *Ifng*^*+/+*^ mice treated with anti-IL-10R antibodies (Figs. S4i, 4j). Notably, *Ifng*^*-−/−*^ mice receiving IL-10R blockade presented aggravated signs of injury and markedly delayed regeneration, indicating that IFNγ and IL-10 do not have redundant functions in vivo and that simultaneous disruption of both pathways severely impairs recovery from irradiation-induced intestinal injury (Fig. 4j). We subsequently sought to determine the time window during which both cytokines promote regeneration. First, we found that in vivo neutralization of IFNγ starting on day -1 prior to ABI led to increased acute weight loss and delayed regeneration (Figs. 4k, S4j–k). Next, we administered dual cytokine blockade beginning at the nadir of weight loss on day 6 following ABI and observed delayed recovery from weight loss in both IFNγ-blocked and IL-10R-blocked WT animals (Figs. S4j, k, 4k). The recovery of the mice that received dual cytokine blockade was the most severely impaired. Taken together, these findings indicate that IFNγ not only contributes to the acute response to irradiation-induced injury but also plays a critical role during the later phase of recovery. Moreover, they suggest that IFNγ and IL-10 are capable of promoting intestinal regeneration at time points following fulminant injury.

### T_reg_ cells promote organoid growth via mTORC1 and Myc activation in ISCs

To elucidate the molecular mechanisms underlying our observations, we conducted scRNA-seq of murine SI organoids i) cultured alone, ii) co-cultured with T_reg_ cells, iii) co-cultured with T_reg_ cells in the presence of IFNγ/IL-10R blocking antibodies, or iv) stimulated with rIFNγ and rIL-10 (technical details are provided in the Supplementary Materials, including Figs. S5, S6).

**Fig. 5:**
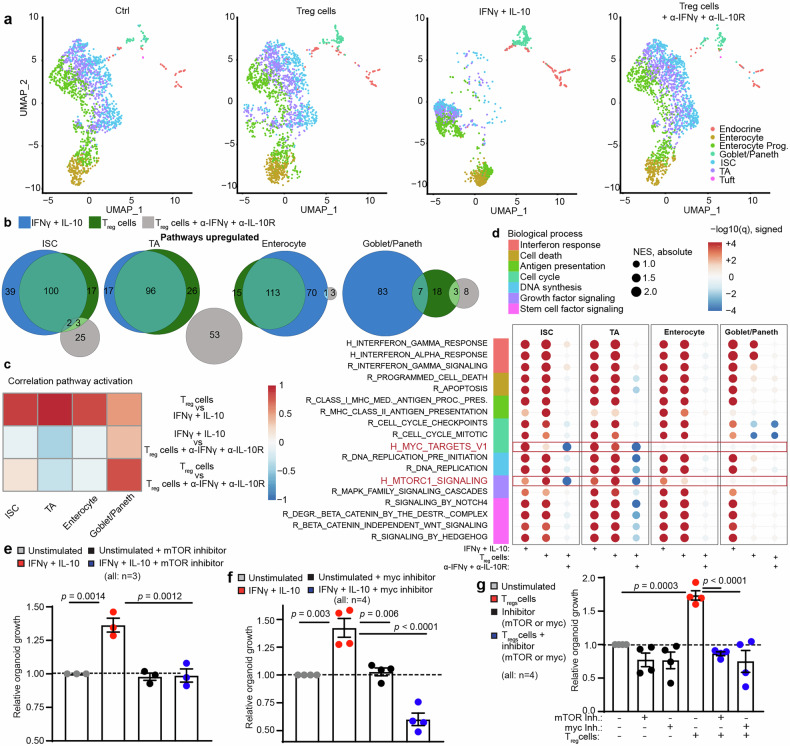
T_reg_ cells promote organoid growth via mTORC1 and Myc activation in ISCs. Murine SI organoids were co-cultured with CD25^high^FoxP3-GFP^+^ T_reg_ cells alone or in the presence of IFNγ and IL-10R blocking antibodies, or were stimulated with rIFNγ + rIL-10 for 4 days, and subsequently analyzed via scRNA-Seq. The data were pooled from 3 independent experiments. **a** Plots of single cells in UMAP space for all experimental conditions, colored by SingleR cell type annotation. **b** Venn diagrams indicating the overlap of upregulated pathways between experimental conditions and control organoids. Only gene sets/pathways significantly upregulated when controlling for an FDR of 10% were considered. **c** Heatmap of Pearson correlation coefficients obtained from correlating GSEA-derived NESs (normalized enrichment scores) between conditions. The values indicate correlations of regulated gene sets/pathway activation between different conditions in the indicated cell types. Only gene sets/pathways significantly up- or downregulated when controlling for an FDR of 10% were considered. **d** Dot plot of the GSEA results of selected pathways/gene sets for different cell types and treatments (vs. control). Dots are colored according to the negative log_10_ of the GSEA q value (FDR), and the sign indicates the direction of the regulation (up positive, down negative). The size of the dots corresponds to the GSEA NES. Gene sets/pathways are derived from the Hallmark (H) and Reactome (R) gene set collections of MSigDB. **e** Relative organoid growth of SI WT organoids ± stimulation with 0.25 ng/mL rIFNγ and rIL-10 and ± the mTOR inhibitor rapamycin (1 µg/mL) or control (DMSO), **f** ± myc inhibition (compound 10058-F4, 100 µM/mL) or control (DMSO), and **g** ± co-cultured T_reg_ cells ± myc inhibition ± mTOR inhibitor. The dotted line represents the growth of control organoids without stimulation. The number of biological replicates (*n*), indicating the number of separate organoid culture experiments, is shown in the figure. The data were analyzed via ordinary one-way ANOVA for multiple comparisons and are presented as the means ± S.E.M.s

Graph-based clustering analysis of cells yielded distinct clusters that emerged prominently after stimulating organoids with rIFNγ plus rIL-10 and also appeared in T_reg_ cell co-culture (Clusters 7, 8 and 12 in the UMAP (Uniform Manifold Approximation and Projection) visualization in Fig. S5a). Notably, this shift was diminished when IL-10 R and IFNγ signaling was inhibited. On the basis of a scRNA-seq reference dataset from the murine intestine,^63^ we identified ISC, transit-amplifying (TA), and enterocyte progenitor cell subsets in clusters 7 and 12. Cluster 8 corresponds to enterocytes and enterocyte progenitors (Fig. 5a, S5b). When clustering at a lower resolution, which yields larger clusters corresponding to a less fine-grained categorization of cells, the less differentiated cells belonging to treatment-related clusters 7 and 12 already emerge as a separate cluster (Cluster 4, Fig. S5c). This indicates that cytokine treatment and co-culture produce a very distinct gene expression pattern in cells at this level of differentiation. On the other hand, the more differentiated cells belonging to treatment-related cluster 8 do not form a separate cluster at lower resolution but rather stay associated with untreated cells (in cluster 1, Fig. S5c). This, together with the observation that there are no shifted clusters for endocrine and goblet/Paneth cells, indicates that co-culture of organoids with T_reg_ cells or combined rIL-10/rIFNγ stimulation induces more pronounced changes in less differentiated cell types, which play critical roles as mediators of intestinal renewal and regeneration. Differentiated cell types, such as enterocytes and enteroendocrine, Paneth and goblet cells, appear to be less affected under these specific conditions (Fig. 5a).

GSEA comparing control and various treatments across different cell types revealed that both co-culture with T_reg_ cells and stimulation with rIFNγ/rIL-10 regulated numerous distinct pathways in intestinal organoids, with strong overlap between the two conditions (Fig. 5b, S5d). In line with this overlap, a high correlation of pathway regulation was observed when Pearson correlation analysis was performed on the basis of the GSEA-derived normalized enrichment scores (Fig. 5c). This effect was prominent in ISCs, TA cells and enterocytes but not in goblet/Paneth cells. In contrast, pathway regulation overlaps or correlations were scarce between, on the one hand, the effects of T_reg_ cells under IFNγ/IL-10 blockade and, on the other hand, T_reg_ cell co-culture or rIFNγ/rIL-10 stimulation (Fig. 5b, c).

In search of the mechanisms of growth stimulation, we detected uniform upregulation of specific pathways in all epithelial cell types, including pathways (interferon signaling, cell death and antigen presentation) that have previously been associated with IFNγ^29,39,64^ and pathways associated with cell cycle regulation, DNA synthesis and growth factor signaling (Figs. 5d, S5e). Intriguingly, we observed a marked upregulation of mTORC1 signaling after stimulation with T_reg_ cells/cytokines, and the effect induced by T_reg_ cells appeared to be more pronounced in ISCs and TA cells than in enterocytes. Additionally, *Myc* signaling was upregulated in ISCs and TA cells under these conditions, whereas no effect was detected in enterocytes or goblet/Paneth cells. Both mTORC1 and *Myc* signaling upregulation was abolished when combining T_reg_ cell co-culture with IFNγ and IL-10 signaling blockade (Fig. 5d). We thus hypothesized that both pathways may play essential roles in ISC/TA cells during T_reg_ cell-mediated intestinal organoid growth stimulation. Indeed, mTOR inhibition with rapamycin (1 µg/mL) or *c-myc* inhibition by compound 10058-F4 (100 µM) abrogated the growth-promoting effects of combined IFNγ and IL-10 stimulation, as well as those of co-cultured T_reg_ cells, on intestinal organoids (Fig. 5e-g).

We conclude that T_reg_ cell-promoted growth of intestinal organoids is mediated by IFNγ and IL-10 and that mTORC1 and c-*Myc* signaling are essential for these effects. On the basis of our results from scRNA-seq clustering and pathway analysis, we assume that the effects arise mainly through direct stimulation of ISCs and cells in the early phase of differentiation (e.g., TA cells) rather than through more differentiated cell types.

### IFNγ and IL-10 compensate for the depletion of epithelial growth factors

In the intestinal crypt, stem cell factors tightly control the proliferation and maintenance of ISCs and epithelial differentiation. Among others, epidermal growth factor (EGF) is a key regulator of proliferation and is upregulated in response to intestinal damage, promoting regeneration.^65,66^ Moreover, the stem cell factor Wnt is fundamental for the maintenance and restoration of the ISC pool.^67^ We speculated that IFNγ and IL-10 might contribute to intestinal regeneration by substituting for EGF and/or Wnt.

**Fig. 6:**
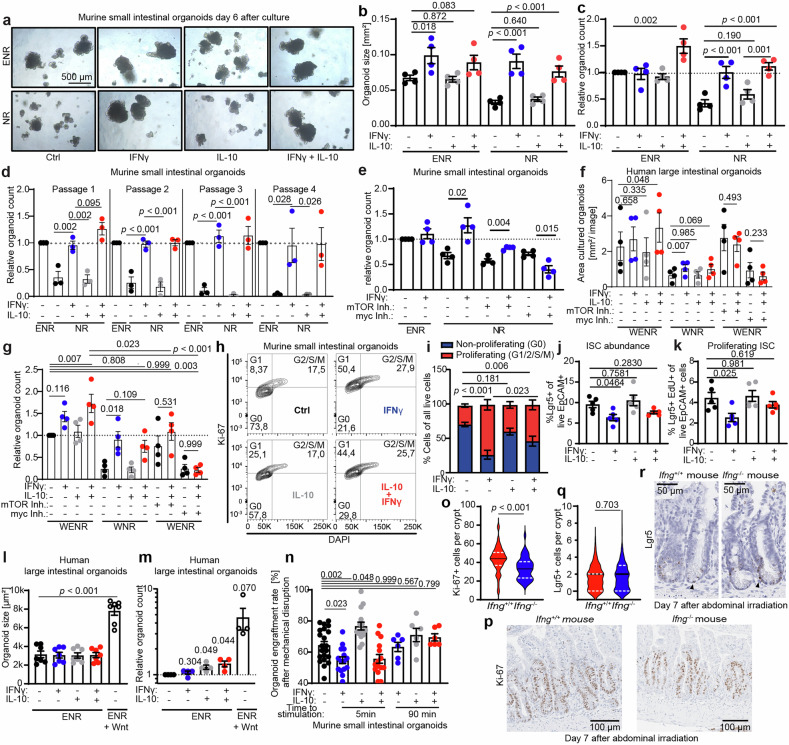
IFNγ and IL-10 compensate for the depletion of epithelial growth factors. Murine SI organoids were cultured under normal growth conditions (ENR) or EGF-depleted conditions (NR + anti-EGF antibody) and stimulated with the indicated cytokines. **a** Representative images, **b** quantification of organoid size on day 6 of culture, **c** relative organoid growth (number of organoids after first passage, compared with control conditions) (*n* = 4 independent experiments), and **d** relative organoid growth during long-term culture and several passages (*n* = 3 independent experiments). **e** Relative organoid growth of murine SI organoids as described above ± mTOR or myc inhibitors (*n* = 4 independent experiments). **f** Human LI organoids were cultured under optimal (WENR) or EGF-depleted (WNR) conditions and stimulated with the indicated cytokines ± mTOR or myc inhibitors. The area of viable organoids per image (used as a surrogate marker for organoid size) was determined on day 6, and **g**) the relative organoid growth (number of viable organoids) was determined after the first passage (*n* = 4 independent experiments). **h** Murine SI organoids were stimulated for 16 h with the indicated cytokines (representative plots). **i** The cell cycle phase was analyzed to distinguish proliferating (G1/2/S/M phase) and non-proliferating (G0 phase) cells (*n* = 6 independent experiments). The proportion of proliferating cells was statistically analyzed. **j** Murine SI organoids were stimulated for 5 days with the indicated cytokines, and the abundance of Lrg5+ ISCs among all viable epithelial cells (EpCAM^+^) and **k** the number of proliferating (EdU^+^) cells among all viable epithelial cells were determined via flow cytometry (*n* = 5 independent experiments). **l** Human LI organoids were subjected to Wnt-depleted conditions (ENR) and stimulated with the indicated cytokines or Wnt. Organoid size on day 6 of culture (*n* = 7 independent experiments). **m** Human LI organoids were cultured under Wnt-depleted conditions and stimulated with the indicated cytokines or Wnt. The number of viable organoids was determined on day 6 of culture (*n* = 4 independent experiments). **n** Murine SI organoids were cultured and mechanically disrupted. One hundred organoids were seeded into culture and stimulated with the indicated cytokines immediately ( < 5 min) or after 90 min. The number of viable organoids was determined on day 6 of culture (*n* = 6–15 engraftment culture wells from 3–6 independent experiments). **o** Analysis of *Ifng*^+/+^ and *Ifng*^−/−^ mice on day 7 after starting ABI (5 × 4.5 Gy/day from day 0 until day 4). The number of Ki-67^+^ epithelial cells within SI epithelial crypt cells was quantified, and **p** representative immunohistochemistry images are shown. Data from 2 independent experiments with *n* = 9 *Ifng*^+/+^ mice and n = 9 *Ifng*^−/−^ mice were pooled, and a total of 131 crypts were analyzed. **q** The number of Lgr5^+^ (Lgr5-GFP^+^) ISCs within small intestinal epithelial crypts was quantified, and **r** representative in situ hybridization images are shown. Data from 2 independent experiments with *n* = 7 *Ifng*^+/+^ mice and *n* = 9 *Ifng*^−/−^ mice were pooled, and a total of 993 crypts were analyzed. Violin plots (**o**, **q**) showing the distribution of values, with medians (solid lines) and quartiles (dotted lines) indicated. All the other data are presented as the means ± S.E.M. *p* values were calculated via two-tailed t tests or ordinary one-way ANOVA for multiple comparisons

To explore whether IFNγ can compensate for the absence of EGF, we cultured murine SI organoids in EGF-depleted NR media (containing Noggin and R-spondin) and simultaneously blocked endogenously produced EGF via the addition of a neutralizing antibody. As expected, this resulted in reduced organoid size and numbers compared with normal growth conditions in ENR media (containing EGF, Noggin, and R-spondin). Stimulation with IFNγ alone fully compensated for the absence of EGF. In contrast, IL-10 alone had no effect, and IL-10 also failed to augment the IFNγ effect (Fig. 6a–c). These effects were durable, as murine SI organoids could be cultured and passaged for several weeks without EGF when stimulated with IFNγ (Fig. 6d). Moreover, we observed that mTOR and c-myc inhibition impaired the growth factor-like effects of IFNγ under EGF-depleted culture conditions (Fig. 6e).

Next, we sought to validate the EGF-compensating properties of IFNγ in human LI organoids. Consistent with our murine data, we observed that IFNγ compensated for the absence of EGF in WNR medium (containing Wnt, Noggin, and R-spondin), as reflected by maintained organoid size and organoid counts after the first passage. In contrast, IL-10 alone did not have such compensatory effects (Fig. 6f, g). Moreover, in the human system, the organoid growth-promoting effects of combined IL-10 and IFNγ stimulation under optimal conditions (EGF-containing WENR medium) were impaired after mTOR and c-myc inhibition (Fig. 6f, g), which is consistent with our findings in the murine SI model (Fig. 5e, f).

We subsequently evaluated the effects of IFNγ on the cell cycle in murine SI organoids. Compared with no treatment, IFNγ stimulation alone induced a rapid transition into the G1 and G2 phases (Fig. 6h, i). IL-10 alone did not significantly impact the cell cycle. However, when combined with IFNγ, IL-10 restricted the IFNγ-induced transition into proliferation, leading to an intermediate level of proliferation compared with that of controls and IFNγ alone (Fig. 6h, i). Therefore, we hypothesized that prolonged IFNγ-induced proliferation might impact the ISC pool. Indeed, five days after the onset of IFNγ stimulation, we observed a reduction in both the overall abundance of ISCs and the fraction of proliferating ISCs (Figs. 6j, k, S7). This depletion was reversed by combined IL-10 stimulation, which had no effect when applied alone (Fig. 6j, k).

Next, we sought to validate the capacity of IL-10 to support ISC retention. Since Wnt signaling is crucial for ISC maintenance in the intestinal crypt,^67^ we cultured highly Wnt-dependent LI human organoids under Wnt-depleted conditions. As expected for the LI organoids, the complete absence of Wnt drastically reduced the size and number of viable organoids after six days of culture compared with those in the Wnt-supplemented cultures (Fig. 6l, m). This decrease in organoid counts was partially reversed by IL-10 stimulation, either alone or in combination with IFNγ (Fig. 6m). However, IL-10 stimulation did not compensate for the reduced organoid size under Wnt-depleted conditions (Fig. 6l), which is consistent with our observation that the organoid size-promoting effects of cytokine stimulation are mediated primarily by IFNγ (Fig. 6a, b, f).

Overall, we conclude that IFNγ promotes organoid growth by enhancing epithelial proliferation but impairs the ISC pool. In contrast, IL-10 counterbalances this effect, preserves the ISC population, and independently supports the survival of highly stressed organoids, such as those cultured under Wnt-depleted conditions. To further investigate the latter, namely the role of IL-10 in supporting organoids under acute stress, we stimulated mechanically disrupted murine SI organoids immediately or, as in previous experiments, 90 min after passaging with IFNγ and/or IL-10 and determined the organoid engraftment rate (number of viable organoids grown). Immediate stimulation with IFNγ reduced the number of engrafted organoids, whereas IL-10 increased this number (Fig. 6n). Notably, under these conditions of acute injury, the negative impact of IFNγ could not be compensated for by co-administration with IL-10 (Fig. 6n). In contrast, delayed stimulation did not induce IFNγ-mediated toxicity but also failed to elicit the beneficial effects of IL-10 (Fig. 6n).

In summary, these results suggest that correctly timed and dosed IFNγ can drive epithelial proliferation and organoid growth. In this context, IL-10 prevents extensive epithelial proliferation and contributes to the maintenance of the ISC pool. The combination of both properties sustainably fosters organoid growth.

Finally, we aimed to confirm the role of IFNγ in epithelial proliferation during intestinal regeneration in vivo. To this end, we analyzed mice on day 7 after the start of irradiation, corresponding to the nadir of weight loss and the time point just before visible recovery began (Figs. 2f, 4j, k). Consistent with our conclusion that IFNγ drives epithelial proliferation, the number of Ki67⁺ proliferating cells in *Ifng*^*+/+*^ mice was greater than that in *Ifng*^*−/−*^ mice (Fig. 6o, p). As expected under these conditions, the abundance of Lgr5⁺ intestinal stem cells remained unchanged, suggesting that IFNγ-driven proliferation occurs without depletion of the ISC pool (Fig. 6q, r).

## Discussion

Excessive intestinal IFNγ^+^ T-cell activation is a hallmark of GVHD pathogenesis and drives other inflammatory intestinal diseases, such as immune checkpoint inhibitor-induced colitis.^68–70^ This may be explained by the capacity of IFNγ to kill ISCs via an on-target effect on IFNγR.^26,29,30^ Here, we discovered an additional regenerative function of IFNγ that becomes apparent in the context of simultaneous IL-10 activation (Fig. S8).

At the cellular level, we found that T_reg_ cells provide both signals (IL-10 and IFNγ) for intestinal regeneration. While IL-10 is an established key cytokine of T_reg_ cell biology, IFNγ-producing T_reg_ cells are less well characterized.^4,8,71,72^ Here, we demonstrate that IFNγ-producing T_reg_ cells (i) promote murine and human organoid regeneration in vitro, (ii) invade the intestinal epithelium almost exclusively after tissue injury in mice, and (iii) locate in close proximity to the epithelial cell layer of mucosal biopsies from allo-HSCT patients but not in healthy controls. The functional relevance of IFNγ^+^ T_reg_ cells is supported by a previous study demonstrating that IFNγ expression by T_reg_ cells is essential for the prevention of murine GVHD.^55^ Our data demonstrate that T_reg_ cells predominantly express IFNγ after adapting to intestinal tissue injury, suggesting the existence of mechanisms that cause intestinal T_reg_ cells to acquire this regenerative function. However, IFNγ produced by T_conv_ cells can contribute to intestinal repair by acting in synergy with IL-10-producing T_reg_ cells. Similarly, a recent study reported that IFNγ produced by CD8^+^ T cells stimulates the growth of bile duct organoids.^73^

At the level of cytokine signaling, the prominent role of IFNγ as a driver of intestinal epithelial regeneration is unexpected,^26,29^ in contrast with the established contribution of IL-10 to the prevention of inflammatory bowel disease.^74–78^ However, unlike the immunomodulatory function of IL-10, which may support intestinal repair through indirect mechanisms (such as reduced damage), its direct effects on the intestinal epithelium are poorly understood.^74–78^ Our results revealed distinct growth factor-like properties of both cytokines, which, when combined, potently promoted organoid growth. Consistently, previous reports have shown that IFNγ mediates IL-10 receptor induction in epithelial cells, suggesting possible interactions.^79^ In line with these findings, dual impairment of IL-10 and IFNγ signaling in vivo significantly aggravated intestinal tissue injury and resulted in markedly prolonged weight loss following injury in vivo and promoted regeneration of the ISC compartment in vitro. While it is known that high doses of IFNγ are toxic,^26,29^ we discovered that even lower levels of IFNγ can be detrimental to the injured ISC compartment (e.g., after mechanical destruction) but strongly promote the proliferation and growth of established organoids, even compensating for EGF depletion. This observation aligns with emerging evidence that IFNs drive intestinal stem cell proliferation in models of intestinal injury.^80,81^ In contrast, IL-10 ensures protection of the ISC niche after injury but is not sufficient to induce cell cycling, which is consistent with previous reports identifying IL-10 as a key factor in maintaining the ISC pool.^27^ Building on the established knowledge that Wnt stimulation markedly improves organoid formation,^82^ we found that IL-10 compensates for Wnt depletion. However, organoid growth remains highly dependent on Wnt signaling, and even combined IFNγ and IL-10 stimulation only partially compensates for Wnt-depleted culture conditions. Both EGF and Wnt can be provided by Paneth cells, which are often reduced upon intestinal injury, correlating with GVHD-related mortality.^20,83,84^ We conclude that the reduction in the number of growth factor-producing bystander cells after injury can—at least in part—be compensated for by combined IL-10 and IFNγ signaling. Taken together, our data support the concept that inflammatory cytokine signaling can serve a bridging function during tissue injury to temporarily ensure regeneration, which may be particularly important under conditions of limited growth factor availability. However, further studies are needed to explore in more detail how these cytokines influence signal transduction in different epithelial cell types and how their activation converges with growth factor signaling. In summary, we suggest a bivalent, dose-, timing- and context-dependent (presence or absence of IL-10) model of epithelial IFNγR signaling that can result in cell death but also favors regeneration.

At the molecular level, concurrent IFNγ/IL-10 stimulation or co-culture of T_reg_ cells with intestinal organoids promotes regenerative stimuli via mTORC1 and *Myc* signaling, particularly in ISCs and undifferentiated progenitor cells, as our results suggest. This finding is in line with the established role of *Myc* in regulating the epithelial cell cycle and differentiation, as well as emerging evidence that the IFNγ-STAT1-Myc axis promotes intestinal epithelial proliferation in allo-BMT models.^81,85^ Consistently, we observed that the activation of IFNγR on intestinal epithelial cells strongly promoted epithelial proliferation. The proposed pro-regenerative function of mTORC1 is in line with previous reports showing that its activation is critical for compensating for damage after radiation or heat exposure.^86–88^ Along these lines, mTORC1 activation has been linked to different aspects of stem cell renewal and proliferation.^89^ However, mTOR hyperactivation in intestinal epithelial cells can also trigger necroptosis, intestinal inflammation, barrier dysfunction and cancer.^90^ Moreover, IFNγ/mTORC1 signaling controls cell death of both undifferentiated (e.g., ISC) and differentiated intestinal epithelial cells (e.g., Paneth cells).^27,29,42^ Thus, the signaling strength of the mTOR pathway and/or co-activated pathways may tip the balance between tissue damage and regeneration. Our results refine this concept by demonstrating that IFNγ/mTORC1 activation stimulates ISC-mediated growth and repair in the presence of IL-10. Although we observed the most potent mTORC1 activation in ISCs and TA cells and no activation in differentiated bystander cells such as Paneth cells, we cannot formally exclude that its activation in differentiated enterocytes may contribute to enhanced epithelial regeneration. Nonetheless, it is reasonable to assume that ISCs and TA cells are particularly responsive to T_reg_ cell or IFNγ/IL-10 stimulation, which is further supported by our clustering of single cells obtained via scRNA-seq. The stronger shift observed for less differentiated cells than for differentiated cells upon stimulation reflects a more substantial change in gene expression patterns.

Our general conclusion that T_reg_ cells promote intestinal regeneration is based on research accomplishments of the last two decades. However, T_reg_ cell-mediated tissue protection is often explained by tolerance-inducing and immunosuppressive functions, which restrict inflammation and thus create a favorable environment for regeneration.^4^ Yet, direct links between T_reg_ cells and tissue repair remain scarce.^13,91,92^ Only a few landmark studies have shown that T_reg_ cells are able to directly induce regeneration via the release of amphiregulin or jagged 1.^7,11,12^ In the context of intestinal damage induced by allo-HSCT, T_reg_ cells were shown to restrain donor T_conv_ cell activation and proliferation in lymphoid organs and thus reduce damage to the gut.^4,70^ However, beyond this effect, the transfer of T_reg_ cells results in enhanced signs of intestinal regeneration after allo-BMT.^93^ Additionally, T_reg_ cells can increase ISC stemness, help maintain the ISC niche in vivo, and foster intestinal regeneration via IL-10 activation during helminth and bacterial infections.^27^

Our findings have translational implications. Using ruxolitinib to treat GVHD in mice, we were able to increase the proportion of intestinal epithelial T_reg_ cells, which was accompanied by enhanced regeneration of the ISC compartment. This finding is in line with our previous report of improved T_reg_ cell differentiation in ruxolitinib-treated mice after allo-BMT.^59^ Ruxolitinib is a JAK1/2 inhibitor that inhibits IFNγR/IL-10 R signaling, which may be incompatible with cytokine-mediated epithelial regeneration. However, therapeutic JAK inhibition in vivo most likely does not completely block epithelial cytokine signaling. Consistent with these findings, ruxolitinib showed the greatest potential to promote intestinal regeneration in mice transplanted with high numbers of allogeneic T cells and presumably potent residual IFNγ activation. We hypothesize that enhanced ruxolitinib-promoted epithelial T_reg_ infiltration paves the way for optimal regenerative responses.

The implications of our findings extend beyond T-cell-mediated diseases, as (i) mice with impaired IFNγ or IL-10 signaling exhibited delayed intestinal regeneration following abdominal irradiation, resulting in severe defects in the context of combined deficiency. Moreover, (ii) T_reg_ cells and IFNγ/IL-10 stimulation promoted the growth of intestinal organoids after irradiation as well. Notably, a previous report revealed that the treatment of mice with recombinant IFNγ prior to total body irradiation resulted in aggravated intestinal injury.^26^ Taken together, these findings i) broaden the proposed regenerative mechanisms of IFNγ/IL-10 to non-immune cell-mediated forms of tissue damage and ii) highlight context-dependent functions on the basis of timing, signaling strength and concurrent factors.

Our study has several limitations. Thus, we provide several lines of evidence that IFNγ and IL-10 are critical for promoting intestinal regeneration and that T_reg_ cells are able to provide both signals. However, to what extent other cellular sources of IFNγ and/or IL-10 contribute to intestinal regeneration by stimulating the same epithelial pathways currently remains unclear. While we provide data that T_conv_ cell-derived IFNγ is critical for restoring epithelial barrier function in vivo, we can only speculate about possible non-T_reg_ cell-derived sources of IL-10 (e.g., B cells, type 2 T helper cells, and macrophages). Importantly, we found that rIFNγ/rIL-10 stimulation promoted epithelial regeneration, which was completely independent of the cellular source. Our model suggests that appropriately timed and dosed IFNγ signaling, particularly in combination with IL-10, supports epithelial regeneration. The cellular sources of these cytokines are likely context-dependent and may vary across disease settings. While our data indicate that T_reg_ cells are capable of providing both IFNγ and IL-10, they are unlikely to be the sole contributors to this regenerative pathway. Further studies are needed to elucidate how IFNγ receptor activation can be precisely regulated to promote tissue repair while minimizing the risk of epithelial toxicity. Finally, we propose a model in which ruxolitinib promotes enhanced intestinal T_reg_ cell differentiation and thereby facilitates tissue repair; however, the broader immunomodulatory effects of JAK1/2 inhibition should be carefully considered when interpreting the corresponding experimental outcomes.

In conclusion, our study provides a new perspective on how the ISC niche responds to immune or non-immune cell-mediated injuries and integrates different cytokine signals to direct the fate of the intestinal epithelium toward tissue destruction or regeneration. While high-dose IFNγ or immediate IFNγ activation during injury is invariably toxic, low-dose IFNγ combined with IL-10 stimulation has a marked stimulatory effect on repair. Tissue-infiltrating T_reg_ cells can simultaneously provide both cytokines to achieve this favorable effect.

## Methods

### Human studies

Human studies were approved by the local authorities (Ethics Commission of the Technical University of Munich, School of Medicine, study number 458/17 S and the Ethics Commission of the University of Regensburg 14-101-0047). These studies included male and female patients regardless of sex.

### Mice

The animals were housed in specific pathogen-free (SPF) animal research facilities and were monitored for pathogens according to FELASA recommendations. The animal studies were approved by the local regulatory authorities. C57BL/6 J (H-2kb) and BALB/c (H-2kd) mice were purchased from Janvier Labs (France). Male or female mice were between 6 and 12 weeks of age at the onset of the experiments. IFNγ-deficient mice (*Ifng*^*−/−*^*)* (C57BL/6 J) and IFNγ receptor-deficient mice (*Ifngr1*^*−/−*^*)* have been described previously.^94,95^ FoxP3-EGFP mice were kindly provided by Bernard Malissen and have been described previously.^96^ Lgr5-GFP C57BL/6 J mice were obtained from Jackson Laboratory (RRID:IMSR_JAX:008875). The transgenic mice were co-housed with age- and sex-matched WT controls after weaning for a period of 4–6 weeks before starting *the* in vivo experiments.

### Induction of GVHD after allo-BMT and treatment with ruxolitinib

Induction of GVHD after allo-BMT with myeloablative TBI in a major mismatch (H-2kd/ H-2 kb) GVHD mouse model was performed as previously described.^97^ Treatment with ruxolitinib was performed as previously described.^59^ Briefly, BALB/c recipients were intravenously injected with 5 × 10^6^ allogeneic (C57BL/6 J donor mice) T-cell-depleted BM cells directly after myeloablative TBI with 2 × 4.5 Gy (medium dose). In some experiments, the mice received 2 × 4.0 Gy (low dose) or 2 × 5.0–5.5 Gy (high dose). Co-transplantation of allogeneic T cells was typically performed with 0.5 × 10^6^ purified allogeneic donor T cells (medium dose). In some experiments, the mice received 0.1 (low dose) or 1.5–2.5 × 10^6 ^T cells (high dose), as indicated in the figure legends. Details are described in the Supplementary Materials.

### Crypt isolation

The isolation of intestinal epithelial crypts was performed as previously described.^24^ Briefly, small intestines were cut longitudinally, washed and incubated in 10 mM ethylenediamine-tetraacetic acid (EDTA) for 25 min (4 °C) to dissociate the crypts. The supernatant containing the crypts was collected.

### Murine organoid culture (generation of organoids for further experiments)

Crypts were suspended in liquefied growth factor-reduced Matrigel (Corning) (33% ENR-medium or PBS; 66% growth factor-reduced Matrigel) at 4°C and plated in delta-surface Nunc 24-well plates in 30 μL drops, each containing approximately 200 crypts. After the Matrigel drops were polymerized, 500 µL of complete crypt culture medium was added to the small intestine crypt cultures. ENR medium was prepared as follows: advanced DMEM/F12 (Life Technologies), 2 mM L-glutamine (Sigma), 10 mM HEPES (Life Technologies), 100 U/mL penicillin, 100 μg/mL streptomycin (Life Technologies), 1.25 mM N-acetyl cysteine (Sigma), 1x B27 supplement (Life Technologies), 1x N2 supplement (Life Technologies), 50 ng/mL mEGF (PeproTech), 100 ng/mL rec. mNoggin (Peprotech), and 10% human R-spondin-1-conditioned medium from hR spondin-1-transfected HEK 293 T cells. All the plates were incubated at 37 °C / 5% CO_2_ and the medium was replaced every 2–3 days. After 7 days, the organoids were passaged via mechanical disruption with a 5-mL syringe and an 18-G needle.

### Ex vivo murine organoid culture (assessment of ex vivo organoid regeneration)

The isolation of murine small intestinal crypts was carried out as described above (section “Murine Organoid Culture”). Crypts were isolated on day 7 after allo-BMT during acute intestinal tissue injury and counted, and organoid culture was started with a defined number of crypts (*n* = 200/drop). At least two drops per mouse were generated (technical replicates). The number of viable organoids was counted on day 3 after seeding, divided by the number of crypts used, and reported in the figures as a percentage of initial crypts (x/200 × 100%). Figure S1f illustrates the experimental setup.

### Human organoid culture

For human organoids, healthy tissue from colon resections of colorectal cancer patients was used. The experiments were performed in a similar manner to the murine experiments but used specific human culture conditions, which are described in detail in the Supplementary Materials.

### Co-culture of organoids and allogeneic T cells

PBMCs from healthy human donors were isolated with Biocoll cell separation solution. T_reg_ cells were then isolated from PBMCs via the CD4^+^CD25^+^ Regulatory T Cell Isolation Kit (Miltenyi Biotec) according to the manufacturer’s protocol. Murine T cells were isolated from C57Bl6/J (*Ifng*^+/+^ or *Ifng*^−/−^) splenocytes with a CD4^+^CD25^+^ Regulatory T-Cell Isolation Kit (Miltenyi Biotec) according to the manufacturer’s protocol. CD4^+^CD25^+^ cells were used as T_reg_ cells, and CD4^+^CD25^-^ cells were used as conventional T cells. For co-culture, species-matched allogeneic T cells were added to passaged human organoids or passaged BALB/c small intestinal organoids with either human IL-2 (30 U/mL, Peprotec) and Dynabeads human T-activator CD3/CD28 (Thermo Fisher, 2 µL per well) or murine IL-2 (30 U/mL, Peprotec) and Dynabeads murine T-activator CD3/CD28 (Thermo Fisher, 2 µL per well). To prevent direct contact between the organoids and T cells, the plates were slightly tilted with the organoid drop above the T cells. T cells were added at the concentrations indicated in the figure legends. Blocking antibodies (InVivoMAb anti-mouse IL-10R (CD210), InVivoMAb anti-mouse IFNγ, InVivoMAb anti-human IFNγ, all Bioxcell) were added at a concentration of 10 µg/mL at the onset of the co-culture. Recombinant IFNγ (recombinant murine IFNγ, Peprotec) was added at the onset of the indicated co-cultures with IFNγ^−/−^ T_reg_ cells at a concentration of 0.25 ng/mL. After 4 days, the T cells were removed. Images for size determination were taken on day 6, and the size of the organoid area was analyzed in a blinded fashion via the ImageJ-based software Fiji. The organoids were passaged after 7 days and counted on day 3 after passage.

### Stimulation of intestinal organoids

For cytokine stimulation, human or murine organoids were seeded. After 24 h, 0.25 ng/mL or 5 ng/mL recombinant IFNγ (recombinant murine IFNγ or recombinant human IFNγ, both from Peprotec), 10 ng/mL recombinant IL-10 (recombinant human IL-10 or recombinant murine IL-10, both from Peprotec) or a combination of both were added to the culture. The concentrations used are based on the literature for IL-10 and on our own results for IFNγ (Fig. S4a).^27^ The mTOR inhibitor rapamycin (1 µg/mL, InvivoGen) or the c-myc inhibitor 10058-F4 (100 µM/mL, Sigma‒Aldrich) was added 6 h after organoid seeding. The cytokines and inhibitors were removed after 4 days. Images for size determination were taken on day 6, and the area of the organoids was analyzed in a blinded fashion via the ImageJ-based software Fiji. The organoids were passaged after 7 days and counted on day 3 after passage.

For organoid stimulation over several passages, the organoids were passaged, and their number after disruption was evaluated. These numbers were then used to calculate relative organoid growth. Afterward, the organoids from all the conditions were reseeded at a density of 80 organoids per well or at the highest possible density if organoid growth was compromised due to the culture conditions.

### Organoid engraftment experiments

Organoids were cultured and passaged as described above (murine organoid culture). After mechanical disruption of the grown organoids, the newly seeded cells were directly stimulated with 0.25 ng/mL rIFNγ and/or 10 ng/mL rIL-10.

### Abdominal irradiation

Specific anatomic regions of the mice were irradiated as previously described.^98^ Details are described in the Supplementary Materials.

### Irradiation of intestinal organoids and co-culture with T_reg_ cells or cytokine stimulation

Intestinal organoids were cultured and passaged as described above and irradiated via previously described techniques.^99^

### Statistical analysis

GraphPad Prism was used for statistical analysis. The statistical tests used for all individual experiments are indicated in the figure legends. Survival was analyzed via the log-rank test. A normal distribution was assumed, and differences between the means of two experimental groups were analyzed via an unpaired t-test. Two-sided tests were used. In specific cases, as indicated in the figure legends, one-tailed t tests were applied when the experimental design was based on prior results and conclusions that justified a directional hypothesis (explanations are provided in the corresponding figure legends). We did not assume a normal distribution in ex vivo organoid cultures or ex vivo scRNA analyses, which clearly revealed non-normally distributed patterns, and the Mann–Whitney U test and Kruskal‒Wallis test were used. We used ordinary one-way ANOVA and the Kruskal‒Wallis test for multiple comparisons and performed Dunnett’s test for multiple-test corrections. Multiple testing corrections were omitted in individual experiments that consisted of multiple previously investigated experimental conditions. P values are shown in the figures. Further details, including the statistical and methodological details of the scRNA-Seq and bulk tissue RNA-Seq analyses, are provided in the Supplementary Materials. The numbers of animals per group (n) and/or number of co-culture experiments (n) are depicted in the figures. We did not perform a sample size calculation prior to initiating the experiments but instead determined group sizes on the basis of published literature and our previous experience with similar experimental setups in animal research.

## Supplementary information

Supplementary Materials

Table S1

Table S2

Table S3

## Data Availability

All sequencing data are publicly available at the time of publication. Single-cell RNA-Seq data from murine organoids were deposited at the Gene Expression Omnibus (GEO) (accession number: GSE252335). The scripts used for scRNA-Seq analysis have been deposited on GitHub (https://github.com/lit-regensburg/scRNA_intest_org_co-culture/). RNA-Seq. Data from bulk murine tissue were deposited at the European Nucleotide Archive (ENA) (accession number PRJEB71507). The processed RNA-sequencing data and lists of differentially expressed genes are available in the Supplementary Materials (Tables S1–S3). This publication includes additional analysis of scRNA-Seq data of human samples that were previously described^62^ and were deposited at GEO (accession number: GSE234357) and previously described^60^ murine data (GEO accession number: GSE223798). Any additional information is available from the corresponding authors upon reasonable request.

## Materials availability

This study did not generate new unique reagents.
